# How do reminder systems in follow-up screening for women with previous gestational diabetes work? - a realist review

**DOI:** 10.1186/s12913-021-06569-z

**Published:** 2021-06-01

**Authors:** Jane Hyldgaard Nielsen, G. J. Melendez-Torres, Torill Alise Rotevatn, Kimberly Peven, Kirsten Fonager, Charlotte Overgaard

**Affiliations:** 1 Department of Midwifery, University College of Northern Denmark, Selma Lagerløfs Vej 2, 9220 Aalborg Øst, Denmark; 2 Public Health and Epidemiology Group, Department of Health Science and Technology, Aalborg University, Niels Jernes Vej, 14, 9220 Aalborg Øst, Denmark; 3 Clinical Nursing Research Unit, Aalborg University Hospital, Aalborg, Denmark; 4 DECIPHer, Cardiff School of Social Sciences, Cardiff University, Cardiff, Wales; 5 Peninsula Technology Assessment Group, University of Exeter Medical School, Exeter, UK; 6 Florence Nightingale Faculty of Nursing, Midwifery & Palliative Care, Department of Child and Family Health Nursing, Kings College London, London, UK; 7 Department of Social Medicine, Aalborg University Hospital, Aalborg, Denmark; 8 Department of Clinical Medicine, Aalborg University, Aalborg, Denmark

**Keywords:** Gestational diabetes mellitus, Type 2 diabetes, Follow-up screening, Reminder, Health prevention, Health research, Complex interventions, Knowledge translation, Evaluation, Realist review, Context-mechanism-outcome configurations, Critical realism

## Abstract

**Background:**

Women with previous gestational diabetes have an increased risk of developing type 2 diabetes later in life. Recommendations therefore urge these women to participate in follow-up screening, 4–12 weeks postpartum and every 1–3 years thereafter. We sought to theorize how reminder interventions to support early detection of diabetes work, for whom, and in what circumstances.

**Methods:**

We used a method informed by realist review and synthesis. A systematic, iterative search in six electronic databases (PubMed, MEDLINE Ovid, The Cochrane Library, CINAHL, EMBASE) had a primary focus on experimental intervention studies and included additional information in relation to identified intervention studies. Analysis inductively identified context-mechanism-outcome configurations present in the evidence.

**Results:**

We located 16 articles eligible for inclusion. A cross-case comparison identified seven grouped context-mechanism-outcome configurations leading to intervention mechanisms relating to changes in women’s reasoning and behavior. Configurations were thematically ordered in relation to Systems Resources, Women’s Circumstances, and Continuity of Care. These were mapped onto a socio-ecological model and discussed according to identified middle-range theories.

**Conclusion:**

Our findings adds to the body of evidence, that reminders have the potential to be effective in increasing participation in the recommended follow-up screening. Our study may assist researchers and policy and decision makers to analyze and judge if reminders are feasible and/or likely to succeed in their specific context. Further research into the perspective of socially disadvantaged and overweight women is needed to avoid unintended consequences such as social inequality in service use and stigmatization in future programs.

**Supplementary Information:**

The online version contains supplementary material available at 10.1186/s12913-021-06569-z.

## Background

Gestational Diabetes Mellitus (GDM) is a rising health concern, globally affecting 1 in 7 births in 2017, and mainly associated with increased weight and age in pregnant women [1]. GDM has severe implications for women and children across the life course [1–3], most notably increased risk of developing type 2 diabetes (T2DM) later in life [3–5]. Around 50% of women with GDM will develop T2DM within 5 to 10 years after birth [6]. It is therefore recommended that women with GDM participate in follow-up screening. In Denmark, guidelines suggest follow- up screening 4–12 weeks postpartum and every 1–3 years thereafter [7].

However, low rates of screening uptake are common [8, 9], particularly decreasing participation 4–6 years after birth (approximately 18%) [9]. This underutilized opportunity for early detection of T2DM results in young undiagnosed women often with less noticeable symptoms, a significant public health challenge as young-onset T2DM is associated with greater morbidity and mortality [2]. Regular screening provides an important opportunity to ensure early detection of T2DM and prevention of later stage complications [2, 3]. In addition, maintaining focus on women’s risk could also allow for lifestyle interventions which could halve the risk of T2DM [1].

A growing body of literature has explored barriers and challenges for participation and shows individual, social, and organizational factors as key reasons for non-participation [10–12]. This implies a multilevel complexity, which is especially challenged by a transition of care and treatment between different healthcare sectors, differences in women’s resources and prioritization, and implementation of guidelines for opportunistic screening in general practice [10, 13, 14].

A systematic review by Jeppesen et al., found that reminder systems for screening for postpartum T2DM were efficient [15]. Reminders could target women and/or health care professionals. For women, reminders included postal, email, telephone calls or text messages. Reminders for health care professionals included electronic reminders implemented in patient record systems. However, Jeppesen et al. also concluded that organization, type and frequency of the reminders should be carefully considered accordingly to the target group, as this appears closely linked to effectiveness and efficiency of the intervention [15]. This raises questions about the applicability of findings to different settings and about the development of well adapted interventions as well as the conditions under which reminder systems interventions should be implemented.

Existing research is, however, limited in explaining how the underlying and contextually dependent mechanisms of reminder systems to ensure early detection of T2DM among women with previous GDM are supposed to produce their intended effect. This explanatory approach, a hallmark of realist evaluation methods, could contribute to a cumulation of knowledge and generation of theoretical models, striving to explain how human change arises from interventions in different settings [16]. Therefore, understandings of not only if reminders are efficient, but also how, for whom and under which circumstances reminders are believed to produce intended and unintended outcomes are important, in order to inform future development and strengthen implementation processes [17].

The task of a realist evaluation is therefore to identify, describe, and later test and refine the conjectured understandings of interactions and relations between mechanisms triggered by intervention resources, contextual factors, and outcomes [16]. This can generate new ideas and thoughts to future program development and implementation processes and is especially important as the effect of public health interventions is believed to unfold over time and develop differently in different contexts [18].

This realist review attempts to develop theory behind future interventions based on reminders which could bridge the gap between evidence of effect and practical implementation [19, 20]. This review has three aims: *1) To explore for whom and under which circumstances reminder interventions are effective, 2) To explore theoretical underpinnings in reminder intervention design, 3) To explore and analyze context- mechanism- outcome configurations that emerged under experimental conditions and delivery settings of reminder interventions.*

## Methods

The reporting of this review was guided by the RAMESES standards by Wong et al., 2013 [21]. Although the steps are presented sequentially within the review process, they are actually overlapping and iterative [21]. As a practical guide, RAMESES has supported a rigorous but open process while ensuring transparency in the final reporting. For further information, this realist review was registered in the PROSPERO database of systematic reviews (http://www.crd.york.ac.uk/PROSPERO/, ID:CRD42019123769).

### Search for evidence

We included evidence, in English or Nordic languages, examining the effect of the use of reminders to increase participation in follow-up screening for women with pregnancy complicated by GDM. As realist program theory takes all the factors involved in determining program success or failure into account, realist reviews include different types of knowledge during evidence gathering [17]. Realist reviews therefore not only use result sections in the primary interventions studies, but all parts of the study (e.g., background documents and authors interpretations) [18]. Other studies providing additional information in relation to the experimental intervention studies were also of interest [22].

#### Selection criteria

##### Inclusion criteria for original research studies

- Population: women with previous GDM
- Intervention: Reminder intervention targeting women with previous GDM and/or health care professionals playing a key role in follow-up screening. Reminders for women were defined as postal reminders, email reminders, or telephone calls/text messages, whereas reminders for health professionals included pop-up electronically implemented reminders/alerts or simple reminders either in paper form posted on medical reports or implemented electronically in the patient registry system. The search for evidence mainly focused on single strategy interventions based on reminders, however multiple strategy interventions were eligible for inclusion if the use of reminders was a significant element of the intervention
- Design: Experimental and quasi-experimental study designs including randomized controlled trials, non-randomized controlled trials, before and after studies
- Outcome: Experimental studies which include one of the recommended tests (OGTT-test, Fasting blood glucose, HbA1c) as outcome
- Additional information (qualitative or quantitative) on implementation processes and intervention deliverers or recipients’ experiences in relation to the already included experimental studies

##### Exclusion criteria for original research studies

- Population: Studies focusing on GDM during pregnancy
- Intervention: Studies where reminders were not a significant element of the intervention
- Design: Studies evaluating outcome without a control group receiving standard care
- Outcome: Studies evaluating outcome without including one of the recommended tests (OGTT-test, Fasting blood glucose, HbA1c) as outcome
- Inability to obtain full text of the article

#### Data sources and search strategy

An initial search enabled identification of relevant index terms and text words used to develop the final search strategy, which consisted of three blocks 1) *Gestational diabetes Mellitus*, 2) *Postpartum follow-up* and 3) *Reminders*. Blocks were combined by use of the Boolean operators OR and AND (OR vertically between synonyms and AND to combine blocks). PubMed, MEDLINE Ovid, The Cochrane Library, CINAHL, EMBASE, as well as the citations databases Web of Science and Scopus, was a part of this strategy. An example of the strategy can be found in the supplemental material (File 1).

A search for unpublished studies was made in the databases Open Grey [23] and Clinicaltrials.gov [24] and the professional network site Research Gate [25], using the same essential keywords. An additional chain search included screening of reference lists within the included experimental studies and a search based on intervention and authors names using Google scholar.

All knowledge identified in relation to each of the included experimental studies was considered as an intervention case, (e.g., a trial protocol and other trial results for the same study are considered one case). In uncertainty, if we had located all additional information related to the included experimental study, authors were contacted. Experimental studies would however still be included in situations where it was not possible to receive an answer from the author.

The search was an iterative process; however, the primary search was made between November 2018 and January 2019, with the last updated searched May 2020.

### Study selection and appraisal

All identified citations were exported to RefWorks ProQuest where duplicates were removed. Titles and abstracts were initially screened based on relevance, and eligibility in relation to the inclusion criteria were assessed for relevant studies in full text. Quality assessments were made using the Cochrane risk of bias tool for RCTs [26] or the ROBINS-I (Risk of Bias in Non-randomized Studies – of Interventions) [27].

Selection, appraisal, and data extraction were carried out and crosschecked by a group of three reviewers. At least two reviewers considered each record, and any disagreements were resolved through discussion, if necessary, with the third reviewer.

### Data extraction

A data extraction sheet was created and tested among the three reviewers. Data on *intervention effectiveness,* determined by the primary outcome of the proportion of women participating in follow-up screening after birth, were extracted on all included experimental studies. Data on the intervention, the context and the actual “working of the intervention” or mechanisms were extracted to identify key elements for the success or failure of an intervention in a specific context information. This is recommended by realist standards [21]. The data extraction therefore also included secondary outcomes (e.g. experience and satisfaction of women and healthcare professionals), the intended intervention (e.g. components, timing, intervention and study population, sample size and intervention theory), setting and delivery context (e.g. location, background rates, socio-economic context, policy system and system of care), intermediate outcomes (e.g. intentions and changes in knowledge, beliefs and attitudes of women and healthcare professionals in the intervention group compared to the control group), implementation outcomes (e.g. issues concerning referral, appointment, contact and performance and analyses of the test) and unexpected or unintended outcomes (e.g. intervention disengagement or resistance in the intervention group compared to the control group).

### Synthesis

A narrative synthesis was conducted regarding the effect of the interventions. With the focus of exploring for whom and under which circumstances reminders were found to be effective, prominent patterns in the data were identified. This allowed us to create a better understanding of the variations previously found in the effectiveness of reminders [15] and to discuss the relevance of this according to the findings of the remaining realist synthesis.

Additionally, an identification of the overarching theories underpinning the included cases was made. The further construction of the analysis entailed an analytic process inspired by the principles of realist synthesis described by Jagosh et al. 2011 [28]. Rationale and definitions of main concepts are illustrated in Table 1 below. The synthesis was conducted in the following iterative and overlapping steps:

1. *Identification of explanatory middle- range theories*. This step entailed looking across all included cases to understand what theories could explain the success or failure of the intervention. The underpinned theories can be both explicitly and implicitly embedded in the descriptions of the interventions [28]. In this review, theories were in some cases explicitly described, but in many cases, they were implicit, and the review team identified the most observable theories explaining how the intervention worked.
2. *Identification of CMO-configurations (CMOc).* This step entailed a deeper understanding of how the intervention can enhance a change in reasoning, alter behavior and lead to an outcome [22], for example, how women in a particular context respond to the reminder and what the outcome of this response is. The process required sorting and analyzing CMOc for each included case, conducting a cross case comparison, and thematically grouping the most essential and strongest substantiated CMOc. This analytic process also included mapping the identified CMOc into a model of different ecological levels.
3. *Discussion of confirmatory and contradictory findings.* The third step included the discussion and final interpretation of findings and was done in the context of the identified middle-range theories when applied to our CMOc, as well as the result of the narrative synthesis and socio ecological understanding. This helped us to support and refine the identified middle-range theories and thereby contribute valuable knowledge and transferable lessons to future development of a program theory. This also includes a better understanding of unintended outcomes and potential harms associated with the use of reminders and environment-focused initiatives.

**Table 1:** Rationale and definition of main concepts

**Middle-range theories**	To identify and understand which theories could explain how reminder intervention work Jagosh et al. 2011 defines middle-range theories as when the theory can retrain its relevance across multiple cases and different context. Thus, it cannot be abstract to the extent that it is disconnected to the actual working of a program, neither can it be so specific that it is only relevant to one case [28].
**CMO-configurations**	To evaluate whether a reminder system increases women’s participation in screening *(O)*, a realist would examine and try to understand the underlying mechanism *(M)* (e.g., information, advice, trust, engagement, motivation) and its contiguous context *(C)* (e.g., demographics, legislation, culture norms) [18] These interactions and relations are defined as Context-Mechanism-Outcome (*CMO-configurations*) [17–19] Dalkin et al. 2015, operationalizes the CMO-configurations (CMOc) formula where intervention resources are introduced in a context in a way that enhances a change in reasoning [22]
**Social ecological theory**	Applying the social ecological theory can provide a framework to increase understanding of a human’s interaction with their physical and sociocultural environments and thereby also the environment’s influence on their reasoning regarding an intervention [46] A social ecological theory could be defined as is integration of person-focused programs with environment-focused initiatives to strengthen physical and social surroundings [47]

## Results

**Fig. 1:**
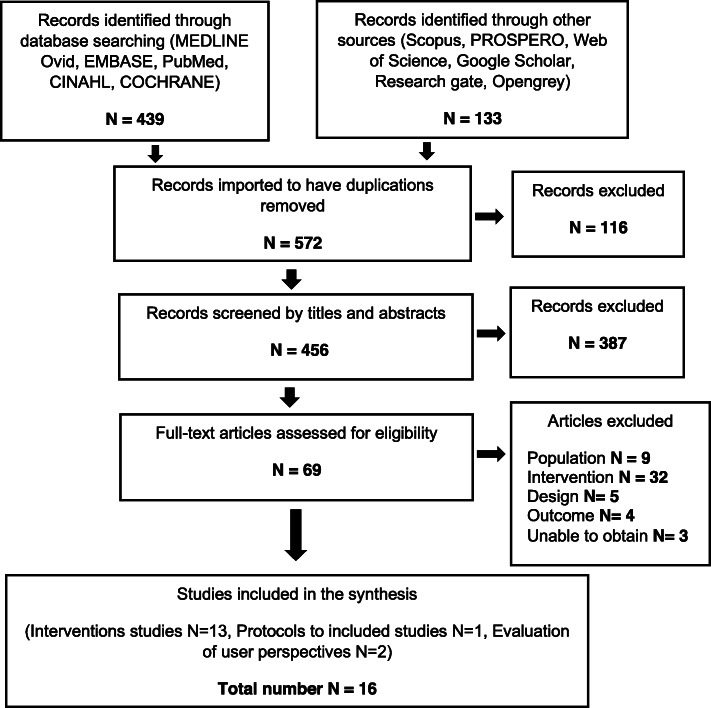
Flow diagram of search results

Thirteen cases were identified, each associated with an intervention based on the use of reminders to support early detection of T2DM among women with previous GDM [29–41]. Three of the 16 included studies were used to inform the intervention cases (one study protocol and 2 surveys which quantitatively examined the user perspective) [12, 42, 43]. Other than this, no relevant references were identified through the information search. Figure 1 presents the search results through a flow diagram.

**Fig. 2:**
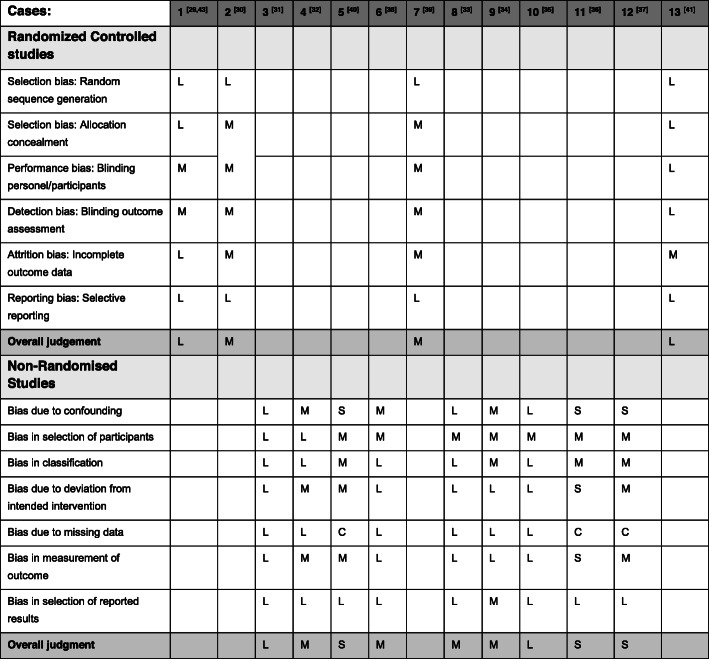
Item level quality assessment of all 13 experimental studies (Risk of Bias: L = Low, M = Moderate, S = Serious, C=Critical)

Of the 13 intervention cases were eight experimental studies (RCTs and pre/post interventions studies) (Case: 1,2,3,5,6,7,10,13) and five observational studies (Case: 4,8,9,11,12). Most of these study designs were found to have low or moderate risk of bias (Case: 1,2,3,4,6,7,8,9,10,13), whereas three studies had a serious risk of bias (Case: 5,11,12). Details for the quality assessment are presented in Fig. 2.

Most intervention cases were delivered in the US (Case: 3,6,7,10) and Canada (Case: 2,5,8,12), with one case from each Australia (Case: 1), Finland (Case: 4), Chile (Case: 11), and the Philippines (Case: 13). All identified studies were in English, and no studies were excluded based on the content of reminders. Table 2 presents study designs, settings, and a short description of the interventions of the cases.

**Table 2:** Characteristics of included studies

Case	Author and year	Study design	Setting /population	Usual care	Intervention components and timing	Outcome	Effectiveness
**1**	**Heatley et al. 2013** **[**43**]**	Protocol	Women’s and Children’s Hospital, Adelaide, Australia. 276 women with GDM were included in the study	A single text message reminder to control group 6 months after birth	*Type reminder:* SMS to women. *Additional components:* None. *Timing*: At birth, 6 weeks after birth, and if no response again three-six months after	*Primary:* OGTT-test. Secondary: Fasting blood glucose, HbA1c. *Time:* 6 months after birth.	**_**
	**Van Ryswyk et al. 2015** **[**29**]**	RCT	**_**	**_**	**_**	**_**	No increase in test: Control 77.6% vs. intervention 76.8%, RR = 1.01, CI: 0.89–1.15
	**Van Ryswyk et al. 2016** **[**12**]**	Survey	**_**	**_**	**_**	Women’s experience	**_**
**2**	**Clark et al. 2009** **[**30**]**	RCT	A tertiary high-risk Obstetric unit in Ottawa, Canada. 220 women with GDM were included in the study	Antenatal clinic visits	*Type reminder:* Postal reminder to women, physicians, or both. *Additional components:* Testing reminders to both women/physicians Timing: 3 months after birth	*Primary:* OGTT-test. Secondary: Other tests. *Time:* Within one year after birth	Test increased: Physicians: OR 8.4 CI: 2.4–28.5, Patients: OR = 8.7, CI: 2.9–25.6, Patients and physicians: OR = 5.2, CI: 1.4–19.6
	**Keely et al. 2010** **[**42**]**	Survey	**_**	**_**	**_**	Women and physician’s experience	**_**
**3**	**Vesco et al. 2012** **[**31**]**	Pre/post	Obstetric department in Washington, USA. 379 women with GDM were included in the study	No reminder for post-partum follow-up	*Type reminder:* Telephone call to women. *Additional components:* Education module for health care providers *Timing*: 3 months after birth and if no response email 3/6 months after birth	*Primary:* OGTT-test ordered and completed. Secondary: Fasting blood glucose. *Time: W*ithin 3 months and 3 months after birth	Test completion increased: from 59.5–71.5%, HR = 1.37; CI:1.07–1.75
**4**	**Korpi-Hyovalti et al. 2012** **[**32**]**	Observational	A central hospital and four rural municipalities in South Ostrobothnia, Finland. 266 women in high-risk-for GDM and their physicians	Women and their physician were included from a lifestyle interventions program during pregnancy	*Type reminder:* Telephone call to women or their physicians. *Additional components:* None. *Timing*: One year after birth	*Primary:* OGTT-test. Secondary: None. *Time:* within study period 2005–2008	Test increased: OR = 13.4, CI: 4.6–38.1, *P* < 0.001
**5**	**Halperin et al. 2015** **[**40**]**	Pre/post	Tertiary high-risk Health Centre in Sunnybrook, Canada. 300 women with GDM were included in the study	Women are provided with a requisition and appointment for screening during pregnancy. Consult notes are send back to the referring physician	*Type reminder:* E-mail to women and fax to family physicians. *Additional components:* Improvements in physicians’ dictations. *Timing*: One months prior to screening test	*Primary:* OGTT-test. Secondary: OGTT-test, Fasting blood glucose, HbA1c *Time:* 6 months after birth. Secondary 12 months after birth	Test increased: from 33 to 44%, *P* = 0.008
**6**	**Soffer et al. 2017** **[**38**]**	Pre/post	Mount Sinai Hospital in New York, USA. 107 women with GDM pre- intervention and 42 post-intervention	Not mentioned	*Type reminder:* Telephone call to women. *Additional components:* Education module for women and health care workers during pregnancy. *Timing*: Before 6 -weeks after birth	*Primary: S*creening visits scheduled, test completion. *Time:* 6-weeks after birth	Test increased: from 17 to 36% *P* = 0.01
**7**	**Zera et al. 2015** **[**39**]**	RCT	Primary care sites in Boston, USA. 850 women with GDM in contact with the primary care site	Screening reminder not visible to providers	*Type reminder:* Message to physicians in electronic health record system. *Additional components:* None. *Timing*: More than 3 months after birth	*Primary:* HbA1c, OGTT-test, and fasting blood glucose. *Time: Dec. 2012*	No increase in test: OR = 1.04, CI 0.79–1.38, *P* = 0.67
**8**	**Shea et al. 2011** **[**33**]**	Observational	Three clinics in Ottawa, Canada. 262 women with GDM were included in the study	Education classes which give information on post-partum screening	*Type reminder:* E-mail to women (A) or Postal reminder/Telephone call to women (B) *Additional components:* A laboratory requisition is included in mail. *Timing*: 3 months after birth	*Primary:* OGTT-test. Secondary: other tests. *Time:* Six months after birth.	Test increased: A: OR = 1.57, CI: 0.66; 3.70. B: OR = 3.10, CI:1.35–7.14
**9**	**Lega et al. 2012** **[**34**]**	Observational	Endocrine Obstetric clinic, Women’s College Hospital in Toronto, Canada 314 women were included in the study, 173 had a checklist on their chart	No checklist was placed in women’s charts during their postpartum visit	*Type reminder:* Checklist in women’s charts during pregnancy to remind physicians to arrange and provide information about follow up screening. *Additional components:* None. *Timing*: between 6 weeks and 6 months after birth	*Primary:* OGTT-test. *Time:* 6 months after birth	Test increased: OR = 2.99, CI: 1.84–4.85
**10**	**Mendez-Figueroa et al. 2014** **[**35**]**	Pre/post	Women and Infants Hospital, New England, USA. 181 women with GDM pre- intervention and 207 post-intervention	Women were routinely informed of screening and a scheduled appointment during pregnancy	*Type reminder:* Telephone call to women. *Additional components:* Information and a pre-scheduled time for screening in pregnancy were provided by an outreach nurse. *Timing*: One week prior to screening 4–6 weeks after birth	*Primary:* OGTT-test. *Time:* 12 months after implementation	Test increased: from 43.1 to 59.4%, HR = 1.59; CI: 1.20–2.12, *P* < 0.01,
**11**	**Olmos et al. 2015** **[**36**]**	Observational	Outpatient clinics in Santiago, Chile. 468 women with GDM were included in the study	Not specified	*Type reminder:* Letter to women *Additional components:* None. *Timing*: In pregnancy	*Primary:* OGTT-test. *Time:* 6 weeks after birth	Test increased: from 32 -76%, *P* = 0.001
**12**	**Peticca et al. 2014** **[**37**]**	Observational	Queensway Carleton Hospital (both secondary and tertiary) in Ottawa, Canada. 542 women with GDM were included in the study	Education module to women during pregnancy	*Type reminder:* Email to women *Additional components:* Laboratory requisition. *Timing*: Within three months after birth	*Primary:* OGTT-test. Secondary: OGTT-test, Fasting blood glucose, HbA1c. *Time:* Up to 12 months after birth. Secondary 12 months after birth	Test increased: Within first year: OR = 1.85, CI: 1.14–3.01, after first year; OR = 2.54, CI: 1.65–3.91
**13**	**Sarmiento et al. 2019** **[**41**]**	RCT	A Public tertiary referral center at a general Hospital in Manila, Philippines. 308 women, mostly from lower income brackets, with GDM were included in the study	A 10-min lecture on screening prior discharge	*Type reminder:* SMS to women *Additional components:* None. *Timing*: Twice a week at 4 weeks, 8 weeks, and 10 weeks after birth	*Primary:* Clinics visit and OGTT-test. *Time:* Within 6 to 12 weeks after birth	No increase in test: adjusted RR 0.98, CI: 0.63–1.52; *P* = 0.932

### Narrative synthesis of the effect of the intervention

Overall, the included cases showed that the use of reminders could be effective in increasing the number of women receiving screening tests postpartum, as ten cases reported a positive effect (Case: 2,3,4,5,6,8,9,10,11,12). Variations were seen in effect with percentage point increases ranging from 11 to 44% among some studies, and variations in odds ratios from 1.85 to 13.4 among other studies. However, three cases reported no such effects (Case: 1,7,13).

The three cases reporting no effect were considered to have a low or moderate risk of bias. However, two of these (Case: 7,13) were performed in a setting where participation in follow-up screening was associated with a cost, which could constitute a barrier for participation. The three studies which were associated with serious risks of bias (Case: 5,11,12) were amongst the ten cases reporting a positive intervention effect. In all these cases, the risk of bias was related to missing data, and especially to lack of available outcome data (Fig. 2). Furthermore, two studies (Case: 2,4) had very wide confidence intervals (Table 1), showing uncertainty around these results.

All interventions differed in terms of overall strategy and type of reminder. Strategies used were either based on a simple strategy, which solely focused on the use of reminder or on multiple strategies, which combined the reminder intervention with other components such as staff training and/or educational initiatives to women diagnosed with GDM during pregnancy. The three cases reporting no effect applied a simple strategy (Case: 1,7,13), reminding women (1,13) or physicians (7). The cases reporting positive effects were split; five cases used multiple strategies (Case: 3,4,5,6,10) and five cases using simple strategies (Case: 2,8,9,11,12).

There was great variety in types of reminders used (e.g. email, phone call, short message service (SMS) etc.) and the target of the reminder (e.g. women or clinicians). Of the cases reporting no intervention effects, two used SMS sent to the women (Case: 1,13), and one used a reminder integrated in health clinicians care systems (Case: 7). In the cases reporting positive intervention effects, the reminder was directed to either the women (Case: 3,4,6,8,10,11,12) or the health clinician responsible for performing screening test (Case: 9), or to both (Case: 2,5). The effective cases used emails, letters by post, telephone calls, fax or a checklist to health clinicians placed on the front of the women’s chart, but not SMS.

Minor differences in the timing of intervention delivery were found between cases reporting intervention effects versus no effects, as almost all cases delivered the intervention in the range of 1 to 6 months after birth and none beyond one year after giving birth. Also, uptake of the oral glucose tolerance-test (OGTT) (primary) and Fasting blood glucose and HbA1c (secondary) were the dominant choices of outcome measures. All cases focused on performing the first screening within one year postpartum for women with previous GDM. Attempts to evaluate long-term compliance with follow-up screening for these high-risk women are therefore not addressed among the included studies.

### Identified overarching theories

All cases were underpinned by a proposition that development of diabetes after pregnancy complicated by GDM can be prevented. The use of reminders as well as the other components (e.g. staff training and/or educational initiatives for women with GDM during pregnancy), draws on the overarching theory that information about risk of T2DM and benefits of screening can lead to important behavior changes, and increase completion rates of follow-up screening of these women postpartum. This theory seemed to be underpinned by social cognition models, perceiving humans as rational beings and based on the belief that change in behavior happens through a change in their cognitive processes, while relying upon a provision of relevant knowledge [44].

Theories underlying the reminder intervention targeted three main areas in the attempt to improve uptake. Some targeted change for the primary health care clinicians responsible for performing screening tests and were based on the beliefs that the use of reminders for clinicians could increase their compliance with guidelines and/or provide a continuous focus on this specific group of high-risk women (Case: 2,3,7,9). Other cases targeted change for women and believed that information about personal risk of developing diabetes and time for screening would lead women to prioritize participation in follow-up screening in an otherwise busy and sleep deprived period of time. Reminders were thus believed to increase women’s motivation and encouragement to participate in screening (Case: 4,5,6,8,11,12,13). Even though it was not explicitly described, this target for change also seemed to be underpinned by theories within behavior change such as theory of reasoned action models, believing that humans are likely to do what they intend to do and are able to rationally, systematically, and logically use information [44].

Finally, some cases were underpinned by understandings of the importance of continuity of care and that a reminder could help minimize the loss to follow up between health care sectors and support continuity in women’s care across sectors (Case: 10,13). One study described this as reminders contributing to decision making processes (Case: 2). While it was not elaborated on, it is well recognized that three types of continuity are required to ensure high quality care: informational continuity; relational continuity; and management continuity [45].

### Thematic overview of the identified CMO-configurations (CMOc)

**Fig. 3:**
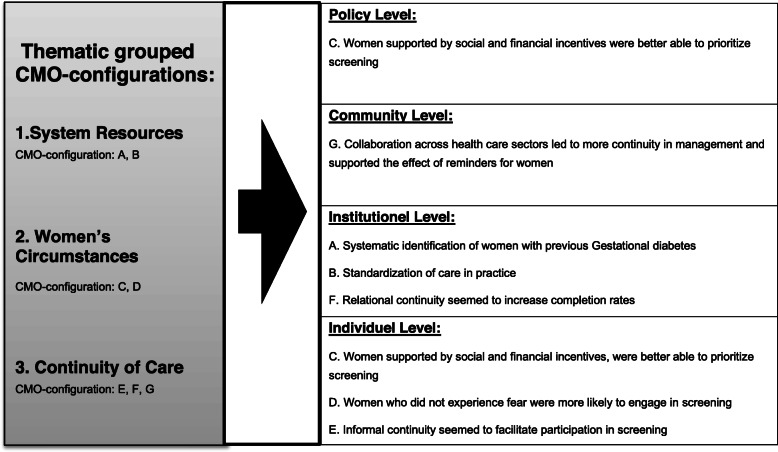
The thematic overview of the identified CMO-configuration and the visualization of how these CMO-configuration map into different ecological systems

The CMOc’s were extracted from each intervention case and described separately. An example is presented in supplemental material (Case: 1, File 2). In the overall extraction process, 108 CMOc’s were identified among the included studies. The emerging CMOc’s were then, through a cross case comparison amongst all 13 cases, consolidated into seven CMOc’s under three thematic headings. The three thematic headings consisted of CMOc’s related to system resources, women’s circumstances, and continuity of care. The CMOc’s were then mapped against different ecological systems, making it clear that resources and reasoning, important for the success or failure of reminders, works across different ecological levels. The process is illustrated in Fig. 3, while the three themes and CMOc’s will be elaborated in the sections below.

#### Theme 1: System resources

##### CMOc A: *Systematic identification of women with previous GDM provided an opportunity to track completion rates and repeat reminders (Case: 3,6,10)*

Some multiple-strategy cases sought to change women’s behavior by tracking completion rates and sending out reminders to women (context). This provided researchers (Case:3,10) and health care professionals (Case: 6) with the opportunity to repeat reminders to women and sometimes actively attempt to minimize practical barriers to participate (mechanism). This constant professional focus on women with previous gestational diabetes seemed to underline the importance of screening to women and to facilitate participation, as it led to increased completion rates (outcome) (Case:3,6,10).

##### CMOc B*: Standardization of care in practice provided clinicians with important information on care and a reminder to order tests (Case: 5,6,7,9)*

Both simple and multiple strategies sought to increase knowledge and remind health care professionals of screening by standardization of knowledge in patient records or by implementing lists of care orders for clinicians (Context) (Case: 5,6,7,9). This provided clinicians with important knowledge of screening recommendations as well as prompted them to order tests during pregnancy or when women came to them in general practice (mechanism). In most cases this led to an increase in completion rates (Case: 5,6,9). However simple strategies depending only on reminders to health care professionals but not to women, implicitly relied on continuity in health care professionals; thus, a simple strategy was not successful in bridging the communication gap between health care sectors and reminding clinicians in general practice alone (outcome) (Case:7).

#### Theme 2: Women’s circumstances

##### CMOc C: *Women supported by social and financial incentives were better able to prioritize screening (Case: 1,5,6,7,12,13)*

Different health care systems, demographic characteristics and resources among women were present in the studies testing reminders. Some women found it hard to find the necessary time, financial and social resources to participate (context) (Case:1,5,6,7,12,13), which led them to focus on proximal factors (e.g., childcare) and prioritize the more present needs of the families (mechanism). This also resulted in lower participation rates among women who were not a part of a public or private insurance policy or had low socioeconomic status (Case: 7,13). Women with a higher socioeconomic status appeared to find it easier to overcome barriers related to financial coverage of tests, childcare, getting off work and found the time needed for OGTT manageable, and thereby prioritized screening (outcome) (Case: 12,13).

##### CMOc D: *Women who did not experience fear were more likely to engage in screening (Case: 4,8,13)*

Women who were obese were less likely to participate in screening (Case: 4,8). Women with family history of diabetes or insulin dependent GDM did however recognize the importance of screening and were more likely to participate (context) (Case: 4,13). They felt that the reminder provided important knowledge which increased their interest in diagnosis, prevention, and future health (Case: 4,13), whereas women with obesity reported a fear of getting diagnosed (mechanism) (Case: 4). This led to an increase in completion rates among some women but a reduced chance of early detection for the women most at need (outcome) (Case: 4,8,13).

#### Theme 3: Continuity of care

##### CMOc E: *Informal continuity seemed to facilitate participation in screening (Case: 2,4,8,12)*

Reminders to women sought to minimize the documented barriers related to women’s uncertainty of their own risk and the effect of screening (context). The reminder therefore provided information of risks as well as benefits of screening. In these cases, the information was similar to the knowledge, education or counseling given during pregnancy and thereby provided recognizable information. This use of information on previous events and circumstances to make current care appropriate for the individual provides informational continuity [45] (mechanism), which led women to overcome the barriers related to uncertainty and resulted in increased completion rates (outcome) (Case:2,4,8,12).

##### CMOc F: *Relational continuity seemed to increase completion rates (Case: 8,9,10,12)*

Some interventions and contexts entailed a known health care team, case-managers, or a personal contact being available to women (context). This allowed health care providers to ease transitions in care and to reduce stress for women in the clinical setting or in the process of transition from one health care sector to another. This type of ongoing relationship between patients and providers connects care over time and creates relational continuity [45] (mechanism). In all cases that supported this type of relational continuity, the reminder was found to increase completion rate (outcome) (Case: 8,9,10,12).

##### CMOc G: *Collaboration across health care sectors led to more continuity in management and support of the effect of reminders for women (Case: 2,3,4,5)*

Clinicians experienced lack of knowledge of their patients GDM diagnoses but agreed on their responsibility to test women, wherefore reminders also targeted clinicians (Case:2,4,5) and some researchers made presentations on this outside hospital settings (context) (Case:3). These interventions provided an attempt to remind clinicians and enable necessary information sharing, as well as create collaboration with the health care professional involved in screening. However, in one case delivery rates were low (Case:4). This type of collaboration ensures that care from different providers is connected in a coherent way and strengthens management continuity [45], as well as led to local anchoring of knowledge (mechanism), which contributed to increased completion rates (outcome) (Case 2,3,4,5).

## Discussion

### Key results

To support and refine the identified middle-range theories and thereby contribute valuable knowledge and transferable lessons to future development of a program, our findings are discussed accordingly to the socio ecological understanding, looking at the findings in a larger perspective focusing on the interplay between individuality and societal structures [44, 46, 47]. Overall, our findings contribute to an understanding of how use of reminders can lead to behavior change, increasing participation in follow-up screening and that continuity of care plays a significant role. Furthermore, our findings suggest that future development of reminder interventions should clarify if environmental changes are needed rather than focus solely on creation of change within individuals which was the primary objective of the identified approaches to behavior change in this study. Our findings add to previous understandings: providing individuals with motivation to change behavior cannot be effective if the environment makes it difficult or impossible to make healthy decisions [46]. In contrast, it should be convenient, attractive, and economically possible to engage in healthy behaviors, where motivation and education can be initiated after [46]. In our findings we were able to draw environment-focused lessons on all socio ecological levels, for future modelling of interventions, including knowledge of unintended consequences in relation to the use of reminders.

***Individual-level*** (intrapersonal and interpersonal) factors, such as informal continuity for women described in our findings, imply that reminders to provide information on risk of diabetes and the importance of screening should preferably build on the same type of information provided during pregnancy. This seemed to contribute to women overcoming barriers related to uncertainty of their own risk and the effects of screening and eased transition between health care sectors. Reid et al., also advocates for this type of informal continuity; hence information is the common thread that links care and makes current care more appropriate [45].

In our findings, women’s perceived options to participate often weighed against interest and focus on more proximal responsibilities e.g. care of family needs. To better explain these mechanisms in women, Dennison et al. describes how most women plan activities around the needs of the newborn, not around the needs of the medical care system, and if these are not perceived compatible, women do not attend screening [14]. Furthermore, adapting to life with a baby, were in previous studies, found overwhelming to some women, whereas feelings of stress, frustration, and tiredness drowned women’s intensions and concern of own health [14].

Overweight and socially disadvantaged women are in our study found to be less likely to participate, which was believed may be related to fear of being diagnosed [32, 33, 37, 41, 48]. This fear could be a result of what previous literature describes as a cognitive process of self-stigma. Self-stigma entails that a person is aware of the stereotypes that describe a stigmatized group (e.g., that increased risk of developing T2DM among overweight people is self-inflicted), agree with these stereotypes and apply these stereotypes to the self [49]. This leads to social mediators, such as reduced self-esteem and self-efficacy that may negatively influence help-seeking behavior and ability to pursue independent living opportunities [49]. Personal empowerment is a parallel positive phenomenon conceived as a mediator between self-stigma and behaviors [49]. Other explanations could be bad experiences related to being diagnosed with GDM [50], or fear of the negative consequences of living with type 2 diabetes [51].

On an ***institutional level***, our findings suggest that relational and management continuity can contribute to increased participation of women for whom care is delivered across different health care sectors (which is often the case). For some, a sustained attention to the risk and benefits of screening or a recognizable or personalized health care person in charge, lead women to overcome barriers related to uncertainty of their own risk and effects of screening. This then streamlined transition between health care sectors. Reid et al. describes elements like a sustained attention on risk and recommendations and personalized care, as care supported by a shared management plan and a relationship between patients and providers which connects care over time [45]. The effect of this is supported by previous studies reporting that consistency in relationships made some women feel that they knew and trusted their clinicians, and generated feelings of being safe, which was a facilitator to participation [14].

Furthermore, as described in our findings, providing necessary information to clinicians and support of collaboration with clinicians, could also lead to local anchoring of knowledge supportive to increasing screening rates. Qualitative research suggests a lack of focus on women’s risk of T2DM and prioritization of follow-up screening by clinicians to be among the reasons for non-participation [13]. Clinicians can play an important role in women’s ability to understand her own risk and affect women’s motivation to attend screening [14]. Previous research findings also suggest that if the patient-clinician relationship is underdeveloped, women ask fewer questions and loose opportunities to learn more. This may lead women to an experience of having the sole responsibility communicating their own medical history and for participation in screening, which in worst case may lead to frustration or anxiety in women [14]. Support in decision making processes could strengthen women’s sense of control and relieve stress in decision [52]. Moreover, literature suggests that introduction of brief decision support interventions (e.g., reminders), can act as a catalyst for a new discourse and help make shared decision making a practical reality in busy clinics, as they convey awareness of existing choices before the clinical encounter [53]. However, according to Reid. et al. systematic sharing of information is not sufficient to ensure informational continuity for women, as the information must be interpreted and actively used by health care providers to create continuity of care [45].

In relation to the ***community level,*** our finding suggests that attempts to enhance collaboration and a clear pathway of knowledge among health care sectors could support clinicians in providing timeliness and continuity in care. However, contextual factors such as logistics also had an impact in women’s decision to participate.

On a ***policy level***, a significant barrier for participation was found in contexts where no insurance policies were in place to cover screening expenses, and as such, reminders had no effect in facilitating screening [41, 48]. Other contextual factors such balancing work obligations, time used for appointments, and transport were also found likely to influence participation in screening [37, 38, 40, 41, 48, 54]. Our finding do not allow for conclusion on whether future intervention modelling should include multiple strategies, combining reminder interventions with other components as e.g., staff training and/or educational initiatives to women with GDM during pregnancy. Hence, the content of the components (information on risk and recommendation) as well as circumstances surrounding these initiatives, was sometimes part of usual care provided in interventions described as single strategies.

Furthermore, due to the heterogeneity in quality and intervention components amongst effective studies, we were not able to determine the best type of reminder e.g., SMS, telephone, or letter/email, and whether the reminder should target women, clinicians, or both. However, in the literature, multilevel interventions are found to be the most effective and sustainable [46]. In addition, no studies were found regarding the effect of reminders on long term follow-up screening of women with previous GDM. This is nonetheless important because participation in follow-up screening is a recurrent recommendation, where previous studies have found declining participation [9]. It is therefore also possible that women’s response and reasoning arising from intervention resources differs in the subsequent years after birth.

### Strengths and limitations

The inclusion of cases was restricted to experimental studies investigating the use of reminders as well as supplement knowledge related to these. Knowledge focusing directly on implementation processes and intervention deliverers or recipient experiences were limited, a known challenge in realist reviews, as primary studies mostly report on outcomes rather than processes explaining how outcomes come about [18]. Only two out of the 13 included cases were enriched with supplementary information (Case: 1, 2). Nevertheless, one notable strength of this study is that in the few cases where the experimental studies indicated that supplementary information existed but was not retrieved during our search, authors were contacted. However, studies were included even if the authors did not confirm that we had retrieved all supplementary information. Also, the studies in serious risk of bias are believed to have provided important knowledge of context and mechanism, wherefore inclusion is a strength within this study. Secondly, the search was based on an iterative, yet comprehensive strategy, and thus we believe that the low amount of supplementary information reflects few attempts to theorize programs.

Thirdly, our search also found studies on women’s experience of possible reasons for not participating in the recommended screening. However, they did not meet the inclusion criteria of this review (no reminder intervention). Nonetheless, this type of literature could have provided information on both context and mechanism important to participation in follow- up screening. Therefore, were two systematic reviews inclusive of these perspectives used in discussion and understanding of our results [10, 14].

### Implications for practice and research

Our findings give some **implications for practice** as it contributes to knowledge of factors important to the effect of reminders, which moves beyond the individual woman. Contextual factors in relation to both physical and social structures, and thereby the circumstances surrounding women, have an influence on the effect of reminders. Reminders have the potential to play an important role in offering women increased continuity of care and shared decision-making processes with heath care providers. These shared decision-making processes should consider how reminders and risk communication could contribute to positive processes of personal empowerment in women. However, decisions on resources and activities included in future programs (e.g., multiple/single strategy, type of reminder and whom the reminder should target), should be carefully considered according to the contextual ability to provide continuity and organization of women’s care and treatment as well as women’s access to screening, e.g. policy level structures to support women and usual care components. Furthermore, needs of collaboration with clinicians, their role and need of knowledge should also be clarified in the process. Last, but most importantly, avoiding unintended consequences such as social inequality in participation and stigmatization of overweight women should be considered ethical reasons to increase effects of reminders.

**Implications for research** include process evaluation and further theorization of women’s own perceptions of risk, experiences, and acceptability of receiving reminders interventions. Women’s perspectives could contribute important knowledge on mechanisms through which the intervention operates and moderates change (including long term perspectives), as well as give knowledge on intervention fidelity and contextual influences on implementation of the intervention [55]. Research into the perspective of socially disadvantaged and overweight women is needed to avoid unintended consequences such as social inequality and stigmatization in future programs.

## Conclusion

Our realist review may assist researchers, clinicians and decision makers to analyze and judge if reminders are feasible and/or likely to succeed in their specific context. Our findings suggest that reminders, in a short-term perspective (within a year after birth), could be effective in providing increased participation in follow-up screening after birth. Furthermore, our findings were found to be both supportive to the identified middle-range theories underpinning reminder intervention and generated input to refinement. As results, environment-focused knowledge with transferable lessons on different socio ecological levels to future development of a program theory based on reminders was discussed. This included understanding of some potential unintended outcomes associated with the use of reminders, such as social inequality in participation and self-stigma. Finally, our realist review contributes to knowledge of important factors in the organization of women’s care and treatment in general, and of the importance of patient-clinician relationship as well as decision-making processes for women involved in follow-up screening after birth.

## Supplementary Information

**Additional file 1.**

## Data Availability

This study contains secondary research of previously published articles hence data are available for the public. Analyses are included in this published article as exemplified in supplementary materials. Further information is available from the corresponding author on reasonable request.

## Abbreviations

GDM: Gestational Diabetes Mellitus
T2DM: Type 2 Diabetes Mellitus
RCT: Randomized Controlled Trail
CMOc: Context/Mechanism/Outcome-configuration
