# Supplementary material for: How do reminder systems in follow-up screening for women with previous gestational diabetes work? - a realist review
Source: BMC Health Serv Res. 2021 Jun 1;21:535. doi: 10.1186/s12913-021-06569-z (PMC8167960; doi:10.1186/s12913-021-06569-z)
Supplement: Supplementary file 1 — Additional file 1. [file 12913_2021_6569_MOESM1_ESM.docx]

**Supplemental material**

**File 1: An example of the applied search strategy**

**PubMed:**

| **1:** | **2:** | **3:** |
| --- | --- | --- |
| **Subject terms:**  "Diabetes, Gestational"  [Mesh] | **Subject terms:**  "Aftercare"[Mesh]  "Postnatal Care"[Mesh]  "Postpartum Period [Mesh]  "Diagnostic Screening Programs"[Mesh]  "Mass Screening"[Mesh] | **Subject terms:**  "Reminder Systems"[Mesh]  "Patient Compliance"[Mesh]  "Telemedicine"[Mesh]  "Delivery of Health Care [Mesh] |
| **Text words:**  (diabet* AND gestational* OR gestationel*) AND pregnan*  "pregnancy diabetes*"  GDM* | **Text words:**  "postnatal care"  "Postpartum follow-up*"  "Follow up program*" | **Text words:**  'reminder system*' |

| **NOTES**: Within each block (1, 2, 3), subject terms and text words combined with OR. (Different possibilities were tried). Followed by a combination between the three blocks with AND. **Final hits: 65** |
| --- |

**File 2: An example of the CMOc extraction sheet**

| **CASE: 1** | **STUDY:** Van Ryswyk et al. 2015; *Postpartum SMS reminders to women who have experienced gestational diabetes to test for Type 2 diabetes: the DIAMIND randomized trial*  **ADDITIONAL MATERIAL:**  Heatley et al. 2013; *The DIAMIND study:* *postpartum SMS reminders to women who have had gestational diabetes to test for Type 2 diabetes: a randomized controlled trail - study protocol*  Van Ryswyk et al. 2016; *Women's views of being screened postpartum for Type 2 diabetes after gestational diabetes: Six-month follow-up for the DIAMIND study* |
| --- | --- |
| **INTERVENTION** | **AIM:** To assess whether an SMS-reminder system for women, after GDM, would increase women’s attendance for an oral glucose tolerance test (OGTT) by six months postpartum  **COMPONANTS:** SMS-reminder to women six weeks after giving birth. If no response, new reminders were sent out again at 3/6 months after birth |
| **CONTEXT** | Women with pregnancy complicated by GDM at the Women’s and Children’s Hospital, Adelaide, Australia. The study population were mostly women between 30-39 years, overweight/or obese, half were Asian, and half were Caucasian, high levels of socio-economic disadvantages, half with secondary education and half with bachelor or more. All most everyone was treated in the public health system |
| **EXTRACTED CMO** | Low participation rates in the recommended follow up screening 6-12 weeks post-partum among women with previous GDM **(C),** an intervention based on short message service (SMS) sent out a remind women to undertake an OGTT **(M).** No increase in attendance compared to the control group were found. However relatively high participation rates were found in both groups (Approximately 77%) **(O)**  Women experience **(C),**   - A lack of time, caused by non-available childcare or a need to focus on the health of the baby, as key barriers for participation - That the test was to long - That they were at low risk of developing T2 diabetes - That they were afraid of being diagnosed with diabetes   Leading women not to prioritize/or hesitate to participate in the recommended screening **(M)**, which meant that **a**pproximately 23% of the study population did not respond to the reminder and had an OGTT done **(O)**  Low participation rates in the recommended follow up screening a reminder system a short message reminder and a national reminder system were established **(C),** Was found preferable to women, especially SMS reminders (M), and increased participation in both groups **(O)**  In Australia it is possible to register for a National Diabetes Services Scheme (if eligible to Medicare), which also sends out reminders to these women 12-16 weeks after expected due date **(C),** providing information on life after GDM and visits for screening at the general practitioner **(M),** leading 64% percent of the women to get screened at their local GP rather than at the hospital **(O)**  In 98% of the cases hospitals had sent out discharge summaries to relevant clinicians **(C),** providing general practitioners with communication on GDM diagnosis and recommendation for follow up screening **(M)**, Likely to have positively influenced the overall high OGTT rate in both the intervention and control group (effect found in other studies) **(O)**  Women in the study were provided information and consent forms by discharge from the hospital and prior to inclusion **(C),** providing knowledge of the risk of type 2 diabetes and benefits of follow up screening among women in both groups **(M),** serving as an unintended component of the intended intervention (SMS short message) raising awareness among all women **(O)** |
| **OUTCOME** | Attendance in the recommended screening, measured on OGTT-test (or fasting blood glucose or HbA1c test) undertaking within 6 months after birth. No increase in attendance compared to the control group were found. |
